# Safety of Tepotinib Challenge after Capmatinib-Induced Pneumonitis in a Patient with Non-Small Cell Lung Cancer Harboring MET Exon 14 Skipping Mutation: A Case Report

**DOI:** 10.3390/ijms231911809

**Published:** 2022-10-05

**Authors:** Liang-Wei Tseng, John Wen-Cheng Chang, Chiao-En Wu

**Affiliations:** 1 Division of Chinese Internal Medicine, Center for Traditional Chinese Medicine, Chang Gung Memorial Hospital, Taoyuan 33302, Taiwan; 2 Division of Haematology-Oncology, Department of Internal Medicine, Chang Gung Memorial Hospital at Linkou, Chang Gung University College of Medicine, 5, Fu-Hsing Street, Kwei-Shan, Taoyuan 33302, Taiwan

**Keywords:** MET inhibitor, tepotinib, capmatinib, next-generation sequencing (NGS), pneumonitis, interstitial lung disease (ILD)

## Abstract

The targeted agents capmatinib and tepotinib provide a new treatment for patients with non-small cell lung cancer (NSCLC) with MET exon 14 skipping mutation (METex14). However, drug-induced pneumonitis is an uncommon but threatening adverse effect found in patients treated with both capmatinib and tepotinib. The safety of treating a patient with a MET inhibitor after drug-induced pneumonitis by another MET inhibitor remains unclear. Here, we present a case of a patient with NSCLC harboring a METex14 who was treated with a standard dose of tepotinib after advanced capmatinib-induced pneumonitis and did not present pneumonitis relapse. Tepotinib may be a safe option when medical professionals consider switching MET inhibitors after patients experience pneumonitis.

**Clinical Practice Points**

- Drug-induced pneumonitis is an uncommon but life-threatening adverse effect of target therapies. The safety of using another MET inhibitor after discontinuing the first remains unknown and such patients have rarely been reported.
- Given the lack of targeted therapies for MET inhibitors, rechallenge with tepotinib after capmatinib-induced pneumonitis could be an option for patients with NSCLC harboring METex14.

## 1. Introduction

MET exon 14 skipping mutation (METex14) occurs in approximately 3% of patients with non-small cell lung cancer (NSCLC) [1] and is often associated with poor prognosis [2]. Targeted agents for this mutation have been introduced and have shown promising effects in recent years. The GEOMETRY mono-1 trial showed that capmatinib had overall response rates of 41% in previously treated patients and 68% in treatment-naïve patients [3]. Furthermore, the VISION trial reported that tepotinib had an overall response rate of 46% in patients with NSCLC harboring METex14, by either tissue or liquid biopsy [4].

Drug-induced pneumonitis, also known as interstitial lung disease (ILD), is an uncommon but life-threatening adverse effect of target therapies [5]. While the GEOMETRY and VISION trials and ensuing clinical studies reported lung-related adverse effects, the incidence of pneumonitis was low (2–6%) and reported cases were limited [6,7,8]. The mechanism of drug-induced pneumonitis is not well known and may result from an MET-dependent or -independent cause, based on the selected epidermal growth factor receptor-tyrosine kinase inhibitor (EGFR-TKI) [5]. Once pneumonitis occurs, the permanent discontinuation of MET inhibitors and corticosteroid treatment have been suggested [6]. Other types of target agents within the same class have been used after drug-induced pneumonitis in patients receiving EGFR-TKI [9,10,11] and ALK inhibitors [12]. However, the safety of using another MET inhibitor after discontinuing the first remains largely unknown and such patients have rarely been reported. 

Here, we present a case of a patient with NSCLC with METex14 who experienced pneumonitis during capmatinib treatment and was safely switched to tepotinib without pneumonitis relapse.

## 2. Case Presentation

**Figure 1:**
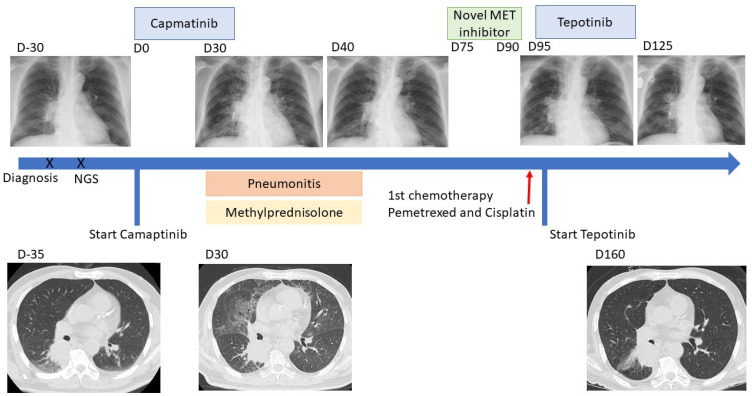
Summary of the clinical course. D0 indicates the first day of capmatinib treatment. Chest computed tomography scans at the time of diagnosis of lung cancer (D-35), at the time of capmatinib-induced ILD (D30), and at nine weeks after starting tepotinib (D160). Chest X-ray at the time of diagnosis of lung cancer (D-30), at the time of capmatinib-induced ILD (D30), at 10 days after corticosteroid use (D40), at the time before starting tepotinib (D95), and at seven weeks after starting tepotinib (D125).

A 69-year-old Taiwanese man without underlying lung disease and smoking history complained of right arm pain for one month. The patient was subsequently diagnosed with metastatic carcinoma based on a specimen from the operation that the patient received for treating a pathological fracture of the right humerus. NSCLC (cT4N2M1b, stage IV, negative PD-L1 expression) with bone metastases was confirmed (Figure 1). Next-generation sequencing by FoundationOne LiquidDx and LC-SCRUM-Japan reported an METex14 mutation, and the patient was started on 400 mg capmatinib twice daily.

Unfortunately, progressive exertional dyspnea and dry cough were reported after one month of capmatinib treatment. Physical examination showed bilateral basal crackles on lung auscultation. The patient’s oxygen saturation was 92% in room air, and chest radiography (CXR) and computed tomography (CT) showed increased interstitial infiltration, which was compatible with drug-induced pneumonitis/ILD (Figure 1). Polymerase chain reaction (PCR) for COVID-19 was negative and there were almost no local cases during that period in Taiwan. Under suspicion of grade 3 capmatinib-induced pneumonitis, capmatinib was discontinued and methylprednisolone was introduced. The patient’s symptoms improved, and pneumonitis subsided on CXR after 7-day methylprednisolone administration (40 mg twice daily for 5 days and then 20 mg once daily for 2 days) (Figure 1). The clinical response was assessed based on the Response Evaluation Criteria in Solid Tumors (RECIST), version 1.1. CT showed stable RLL mass (Figure 1). Capmatinib was permanently discontinued based on the package insert of capmatinib.

Thereafter, the patient participated in a clinical trial of a novel MET inhibitor for two weeks. The patient’s pneumonitis did not relapse, but he was withdrawn from the study because of clinical progression (the metastatic tumor over the right arm progressed rapidly during treatment). No response to a novel MET inhibitor may result from the occurrence of MET resistance or no activity of this compound. Palliative chemotherapy with pemetrexed and cisplatin was immediately administered. Tepotinib, another MET inhibitor, has been reported to be active in treatment-naïve or previously treated patients with METex14 mutation. As this patient may have still benefited from MET inhibitors, tepotinib with a standard dose (one tablet of tepotinib is 225 mg and 2 tables daily is recommended dose) was also prescribed concurrently for one month and discontinued because of clinical progression (right arm tumor). There was no pneumonitis relapse within one month of tepotinib treatment (Figure 1).

## 3. Discussion 

Currently, only two MET-selective inhibitors (capmatinib and tepotinib) have been approved by the U.S. Food and Drug Administration. Owing to the limited treatment options and considerable efficacy of MET inhibitors in clinical studies [3,4], it is necessary to clarify the issues that occur during rechallenge with these drugs in patients who have experienced drug-related intolerable or serious adverse effects. In this case, despite the history of capmatinib-induced pneumonitis, we decided to prescribe another MET inhibitor, tepotinib, while closely monitoring the pulmonary symptoms, oxygen saturation and CXR. After switching to tepotinib, pneumonitis relapse did not occur, as evidenced by normal radiographic examinations of the lungs throughout the treatment course.

In the current report, pneumonitis was found to occur as an uncommon adverse effect of MET inhibitor use for treating NSCLC with uncommon mutations. Therefore, the experience presented in this case report can help inform clinicians about the safety of switching MET inhibitors due to pneumonitis. Whether the dose intensity of MET inhibitors correlates with lung toxicity remains unknown. According to a previous case report, low-dose tepotinib is safe for patients with a history of grade 2 capmatinib-induced pneumonitis [7]. However, corticosteroids were not used due to mild symptoms and a normal oxygen saturation level in that case. The question of whether to restart the full dose of tepotinib in patients with a history of advanced capmatinib-induced pneumonitis (grade 3) which required corticosteroid treatment remained unclear until our report. Notably, this case report addressed this question and found that rechallenge with tepotinib at a standard dose was safe for the patient who had experienced pneumonitis requiring corticosteroid treatment. Although further research is required, our experience offers initial safety data suggesting that tepotinib may be a reasonable option to consider in patients who have recovered from capmatinib-induced pneumonitis.

This case report had several limitations. First, no bronchoscopy study was performed to exclude other causes of pneumonitis. Furthermore, capmatinib-induced pneumonitis was diagnosed based on the rapid improvement of clinical symptoms and CXR after capmatinib discontinuation and steroid therapy initiation. Second, a longer duration of tepotinib therapy should be applied to determine whether pneumonitis relapse occurs after the long-term use of teoptinib. In this case, the patient presented pneumonitis within one month of treatment with capmatinib; therefore, we presume that pneumonitis relapse would occur sooner if an alternative MET inhibitor targeting the same molecule induces the same adverse event [13]. Notably, no pneumonitis relapse occurred during one month of tepotinib treatment, which suggests that tepotinib did not induce pneumonitis in this case.

## 4. Conclusions

We present the case of a patient with NSCLC with a METex14 alteration who experienced capmatinib-induced pneumonitis and safely continued treatment by switching to alternative MET inhibitors, including one from a clinical trial and tepotinib. Given the lack of targeted therapies for MET inhibitors, rechallenge with tepotinib after capmatinib-induced pneumonitis could be an option for patients with NSCLC harboring METex14. Our experience suggests that tepotinib may be a safe therapeutic option for patients who have recovered from capmatinib-induced pneumonitis under close clinical surveillance.
